# Rates and factors for 30-day readmission in patients with substance use disorders during the COVID-19 pandemic

**DOI:** 10.3389/fpsyt.2025.1654157

**Published:** 2025-12-04

**Authors:** Paddy Ssentongo, Jonathan Nunez, Donald Dissinger, Bhavna Bali

**Affiliations:** 1 Division of Infectious Disease and Epidemiology, Department of Medicine, Penn State Hershey Medical Center, Hershey, PA, United States; 2 Department of Psychiatry and Behavioral Health, Penn State Hershey Medical Center, Hershey, PA, United States; 3 Division of Addiction Medicine, Department of Medicine, Penn State Hershey Medical Center, Hershey, PA, United States

**Keywords:** substance use disorder, addiction medicine, hospital readmission, pharmacotherapy, inpatient consultation, health services research

## Abstract

**Background:**

Substance use disorders (SUD) are associated with frequent hospitalizations, premature discharge, and high rates of early readmission. The COVID-19 pandemic further disrupted the continuity of addiction care, heightening the vulnerability of hospitalized patients. Identifying inpatient factors linked to reduced 30-day readmission may guide strategies to improve post-discharge outcomes in this population.

**Methods:**

We conducted a retrospective cohort study of adults with SUD admitted to Penn State Health Hershey Medical Center between 1 January and 30 December 2021, who received addiction medicine consultations. Demographic, social, and clinical characteristics were extracted from the electronic health records. Logistic regression was used to assess the associations between addiction interventions, including educational counseling, pharmacotherapy initiation, and other referrals, and 30-day all-cause readmission, adjusting for age, sex, marital status, insurance, and comorbidities.

**Results:**

Among 561 patients (mean age, 42 years; 62% men), 139 (25%) were readmitted within 30 days. Depression or anxiety was present in 44% of the patients, and 42% reported polysubstance use. Single marital status and the presence of one or more comorbidities were independently associated with higher odds of readmission (adjusted odds ratio [aOR], 2.82; 95% CI, 1.45–5.52; *P* = .002, and aOR, 2.41; 95% CI, 1.06–5.45; *P* = .035, respectively). Compared with those who declined assistance, patients who received educational counseling had significantly lower odds of readmission (aOR, 0.53; 95% CI, 0.31–0.90; *P* = .02), and those who were initiated on addiction-related medications showed a nonsignificant trend toward reduced risk (aOR, 0.62; 95% CI, 0.32–1.21; *P* = .16). In contrast, patients who experienced a self-directed discharge had more than threefold higher odds of readmission (aOR, 3.02; 95% CI, 1.36–6.73; *P* = .007).

**Conclusion:**

In this cohort of hospitalized patients with substance use disorders, inpatient addiction interventions were associated with significantly lower 30-day readmission rates. Educational counseling and pharmacotherapy initiation reduced readmission risk, whereas self-directed discharge was associated with markedly higher odds of readmission. These findings support the integration of structured addiction consultation services to improve post-discharge outcomes and reduce preventable hospitalizations.

## Introduction

Substance use disorders (SUDs) are a public health problem of epidemic proportions in the US that contribute to a myriad of acute and chronic health problems. In 2021, over 100,000 deaths were attributed to drug overdoses (1). SUD is associated with nonadherence to medical care and high utilization of hospital services (2). According to a 2017 study published in the American Psychiatric Association's journal Psychiatric Services, the readmission rates for patients with substance misuse disorders range from 18% to 26% (3). However, initiation of pharmacotherapy for SUD during hospital admission has the potential to improve linkage to outpatient SUD treatment (4–8).

Medications for opioid use disorder (MOUD), including buprenorphine, methadone, and extended-release naltrexone, and medications for alcohol use disorder (MAUD), such as naltrexone and acamprosate, have been shown to significantly improve outcomes when initiated during hospitalization. These pharmacological interventions reduce the risk of overdose, improve engagement in outpatient addiction treatment, and are associated with lower rates of hospital readmission and mortality (6). Randomized trials and observational studies have demonstrated that starting MOUD in the inpatient setting increases post-discharge treatment retention and reduces illicit substance use compared to delayed initiation or referral alone (8). Despite this evidence, hospital-based initiation of addiction medications remains underutilized, often due to workflow barriers, stigma, and a lack of addiction-trained providers.

Hospitalizations stemming from opioid use disorder and its consequences have increased rapidly over the last decade, particularly among those who inject drugs (9) Some of these hospitalizations may be avoidable with engagement in preventive care or improved adherence to medical advice and medication regimens (10, 11). We hypothesized that inpatient addiction-focused interventions, including education, medication initiation, peer support, motivational interviewing, and linkage to outpatient SUD treatment, would be associated with lower rates of return to use, overdose, and rehospitalization. This study aimed to identify patient- and service-level factors associated with readmission during the COVID-19 pandemic, a period that intensified barriers to the continuity of addiction care.

## Methods

### Data source

The inclusion criteria were all individuals over the age of 18 years who were offered inpatient addiction consult services at Penn State Health Hershey Medical Center from 1 January 2021 to 30 December 2021. Records of all included individuals were reviewed retrospectively, and those who died during admission were excluded. Patients were admitted to the general medical, surgical, or ICU units. All data were obtained directly from the Penn State Health electronic health record (EHR; Cerner Millennium) using structured query language (SQL) extraction performed by a data analytics team.

### SUD definition

SUD was defined based on clinician documentation and ICD-10 diagnostic coding within the EHR, including diagnoses of alcohol, opioid, stimulant, cannabis, and sedative use disorders. Prior validation studies have shown that ICD-10 diagnostic codes for substance use disorders have high specificity and moderate sensitivity compared with structured clinical assessments, supporting their use in epidemiological analyses (12, 13).

### Interventions

Addiction interventions were delivered by a small team of addiction-trained clinicians (medical doctors [MDs] and certified registered nurse practitioners [CRNPs]) as part of the Addiction Consultation Service. Interventions were identified using a combination of structured data fields (consult order codes and medication administration records) and standardized note templates used by the Addiction Consultation Service. All interventions were performed during the index hospitalization before discharge. Medication initiation was confirmed using inpatient medication administration records or pharmacy verification logs. Addiction medicine interventions provided during inpatient stay were categorized into three groups:

- Education: Patients were offered counseling and educational support focused on recovery. This included information on community-based programs (e.g., Alcoholics Anonymous, Narcotics Anonymous), relapse prevention strategies, harm reduction practices, and linkage to peer recovery specialists when available.
- Medications: Eligible patients were initiated on evidence-based pharmacotherapy for substance use disorders. These included medications for opioid use disorder (e.g., buprenorphine, methadone, and extended-release naltrexone [Vivitrol]) and medications for alcohol use disorder (e.g., oral or injectable naltrexone, acamprosate, and disulfiram). Medication initiation was overseen by addiction-trained clinicians to facilitate continuity of treatment post-discharge.
- Other Services/Referrals: Patients were connected to higher levels of care or supportive services as appropriate. This included referrals to inpatient or outpatient detoxification programs, residential rehabilitation facilities, skilled nursing facilities, psychiatric services, and other community-based counseling and recovery programs.

Patients could receive more than one type of intervention.

### Covariate definitions

Demographic and clinical covariates, including substance type, race, comorbidities, and insurance status, were extracted from the structured fields in the electronic health record (EHR). Race and ethnicity were based on patient self-reports in the EHR. Comorbidities, including depression, anxiety, chronic pain, and chronic pancreatitis, were defined using ICD-10 codes present in the problem list or encounter diagnoses at the time of admission. Marital status, insurance type, and discharge status (including whether the patient had a self-directed discharge) were recorded in the admission and discharge documentation. Service type (medical *vs*. surgical) and mental health diagnoses were included as covariates in the adjusted analyses.

### Statistical analysis

Data were summarized using means and standard deviations or medians and interquartile ranges for continuous variables with normal or skewed distributions respectively. Categorical variables were summarized using frequency distributions, reporting the numbers and percentages for each variable. Covariates were selected *a priori* based on clinical relevance and prior literature linking demographic, psychosocial, and medical factors to hospital readmission risk rather than data-driven selection procedures.

The primary analysis assessed the association between addiction medicine interventions and 30-day readmission. Interventions were categorized as (1) educational counseling, (2) medication initiation (including office-based addiction treatment [OBAT] medications such as Vivitrol or methadone, (3) other post-service referrals (such as rehabilitation, skilled nursing facilities, psychiatry, or transfer to another hospital), and (4) self-directed discharge. The reference group included patients who declined to receive assistance. Multivariable logistic regression models were constructed using a purposeful-selection approach in which variables selected *a priori* for clinical relevance were entered simultaneously; no automated stepwise or backward elimination was used. The primary outcome was 30-day all-cause readmission (yes/no). The predictor variables included the demographic, psychosocial, and clinical covariates listed above. We assessed multicollinearity using variance inflation factors (VIFs <2 for all variables). As the analysis was exploratory, we did not adjust for multiple comparisons. Only the first hospitalization of each individual during the study period was included. Subsequent encounters were excluded to avoid correlated outcomes and within-person clustering effects. Additionally, only the first readmission within 30 days of the index hospitalization was included per patient to avoid correlated outcomes, and readmissions were captured within the same healthcare system.

Potential confounders included age, sex, race, marital status, comorbidities, anxiety or depression, other mental health disorders, chronic pancreatitis, service type (medical *vs*. surgical), and insurance type. Measures of association were reported as odds ratios (ORs) with 95% confidence intervals (CIs). Kaplan–Meier survival curves were generated to visualize the cumulative incidence of 30-day readmission across post-service intervention groups, and group differences were compared using the log-rank test. These analyses were exploratory and hypothesis-generating because formal sample size or power calculations were not performed. All statistical analyses and figures were generated using the R statistical software (version 4.2.2; Vienna, Austria). Statistical significance was defined as a two-sided P-value <.05.

## Results

### Cohort description

A total of 561 hospitalized adults with substance use disorders were included in the analysis. **Table 1** summarizes the demographic and clinical characteristics of the study cohort. The mean age was 42 years (SD, 14 years), and 62% were men. The majority of patients were White (74%), followed by Black (10%) and Hispanic (5%). Alcohol and opioid use disorders were the most prevalent primary diagnoses, affecting 53% and 42% of the patients, respectively. Polysubstance use was common, with 42% of the cohort reporting it.

**Table 1:** Demographic and clinical characteristics of hospitalized patients with substance use disorders, stratified by 30-day readmission status.

Variable	Overall (n = 561), N (%)	Readmission (n = 139), n (%)	No readmission (n = 422), n (%)
Age, years, mean (SD)	42.1 (13.8)	41.7 (13.5)	43.5 (14.5)
Sex, male	349 (62.2)	85 (61.2)	264 (62.6)
Race			
– White	417 (74.3)	98 (70.1)	319 (75.6)
– Black	56 (10.0)	16 (11.5)	40 (9.5)
– Asian	6 (1.1)	1 (0.7)	5 (1.2)
– Hispanic	28 (5.0)	7 (5.0)	21 (5.0)
– Native American	3 (0.5)	1 (0.7)	2 (0.5)
– Other	49 (8.7)	15 (10.8)	34 (8.1)
Substance misused			
– Alcohol	297 (52.9)	69 (49.6)	228 (54.0)
– Tobacco/Vaping	147 (26.2)	34 (24.4)	113 (26.8)
– Cannabis/Synthetic cannabinoids	101 (18.0)	22 (15.8)	79 (18.7)
– Benzodiazepines/Barbiturates	31 (5.5)	7 (5.0)	24 (5.7)
– Opioids	234 (41.7)	58 (41.7)	176 (41.7)
– Stimulants	106 (18.9)	21 (15.1)	85 (20.1)
– Other	20 (3.6)	4 (2.9)	16 (3.8)
– Polysubstance use	235 (41.9)	51 (36.7)	184 (43.6)
Comorbidities			
– Depression/Anxiety	248 (44.2)	66 (47.5)	182 (43.1)
– Chronic/Neuropathic pain	90 (16.0)	30 (21.6)	60 (14.2)
– Chronic pancreatitis	33 (5.9)	9 (6.5)	24 (5.7)

Values are presented as mean (SD) for continuous variables and n (%) for categorical variables. Stimulants included cocaine and amphetamine-type substances, as recorded in the diagnostic and toxicology records. Other race includes individuals who did not identify as White, Black, Asian, or Hispanic and includes multiracial, Pacific Islander, and those who selected “Other.” Polysubstance use is defined as the diagnosis of two or more concurrent substance use disorders.

Comorbid psychiatric conditions were frequent; 44% of patients had documented depression or anxiety, and 16% had chronic pain syndromes. Medical comorbidities, such as chronic pancreatitis and hepatic cirrhosis, were also observed, reflecting the high clinical complexity of this population.

With respect to post-service interventions provided by the Addiction Consultation Service, 36% of patients received educational counseling focused on recovery resources and harm reduction, 16% were initiated on pharmacotherapy for substance use disorder (including buprenorphine, methadone, or naltrexone), and 17% were referred to additional rehabilitation or psychiatric services. Approximately one quarter (25%) declined any intervention, and 7% experienced self-directed discharge. The overall 30-day all-cause readmission rate was 25% (139 of 561 patients).

### Factors associated with 30-day readmissions

**Figure 1:**
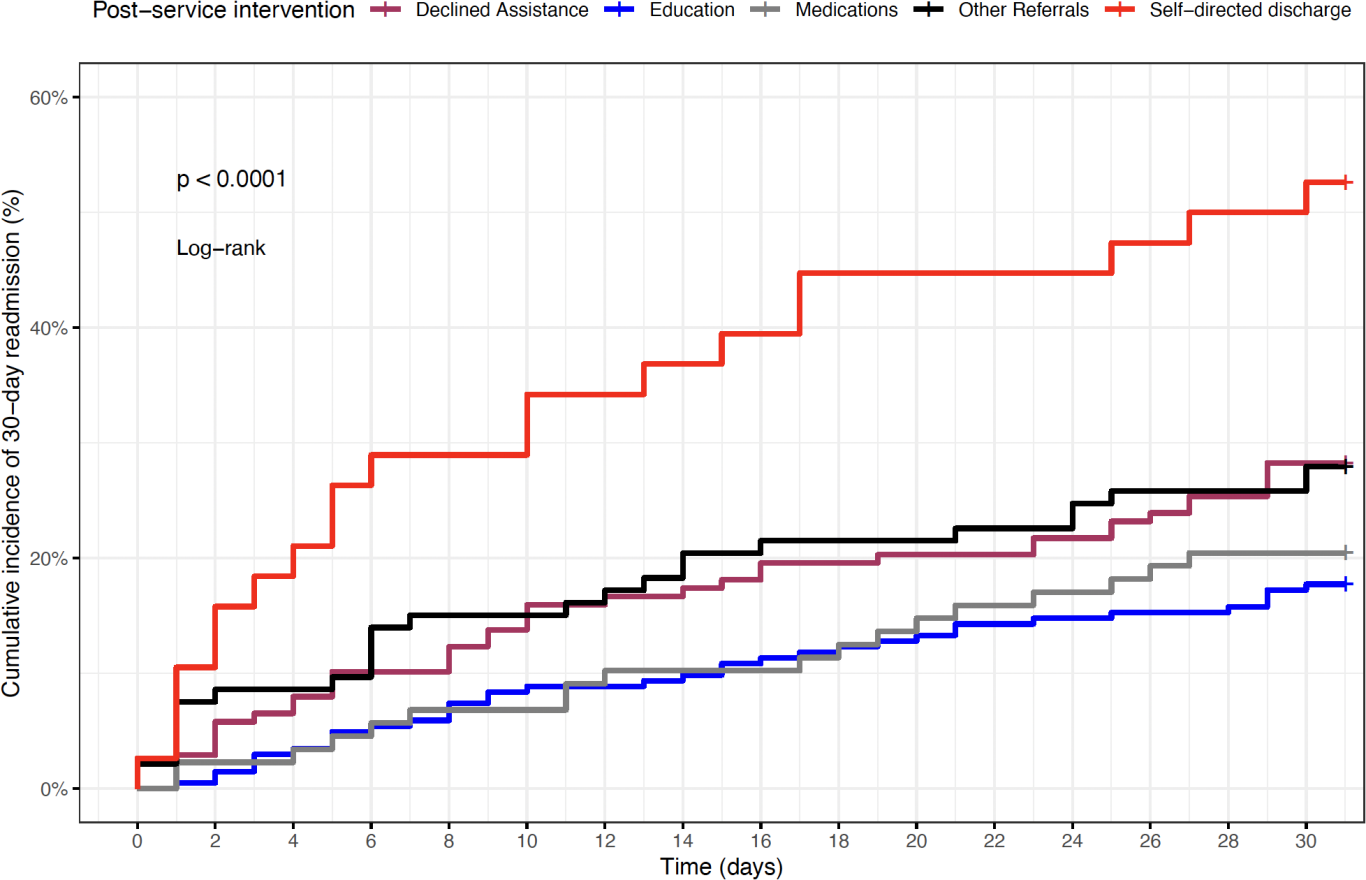
Kaplan–Meier cumulative incidence of 30-day readmission by post-service intervention group.

**Figure 1** displays the Kaplan–Meier cumulative incidence of 30-day readmission, stratified by post-service recommendations from the Addiction Consultation Service. Patients with self-directed discharge had the highest cumulative incidence of readmission, exceeding 50% by day 30 (log-rank P <.0001). In contrast, those who received educational counseling or addiction-related pharmacotherapy demonstrated the lowest readmission risk throughout the follow-up period.

**Table 2** presents the multivariable logistic regression results identifying factors independently associated with 30-day readmission. After adjusting for demographic and clinical variables, patients who received educational interventions had 47% lower odds of readmission than those who declined assistance (adjusted OR, 0.53; 95% CI, 0.31–0.90; p = 0.019). Similarly, initiation of addiction-related medications, including buprenorphine, methadone, and naltrexone was associated with lower but not statistically significant odds of readmission (adjusted OR, 0.62; 95% CI, 0.32–1.21; p = 0.16). In contrast, patients with a self-directed discharge experienced more than threefold higher odds of 30-day readmission than those who declined intervention (adjusted OR, 3.02; 95% CI, 1.36–6.73; p = 0.007).

**Table 2:** Multivariable logistic regression identifying factors associated with 30-day readmission among patients with substance use disorders.

Variable	Adjusted OR	95% CI	p-value
Post-service recommendation			
Educational intervention	0.53	0.31–0.90	0.019
Medication initiation (OBAT/Vivitrol/methadone)	0.62	0.32–1.21	0.159
Other referrals (rehab, SNF, psychiatry, transfer, outpatient counseling)	0.85	0.46–1.57	0.607
Self-directed discharge	3.02	1.36–6.73	0.007
Declined assistance	Reference	Reference	Reference
Comorbidities (≥1)	2.41	1.06–5.45	0.035
Depression or anxiety	1.03	0.66–1.62	0.887
Other mental illness	0.88	0.52–1.49	0.627
Chronic pancreatitis	0.93	0.40–2.18	0.875
Hepatitis or cirrhosis	1.51	0.92–2.46	0.101
Medical service (*vs* surgical)	1.69	0.75–3.82	0.206
Age (per year)	1.02	1.00–1.04	0.013
Sex (women *vs* men)	1.01	0.65–1.59	0.955
White race (*vs* non-White)	0.84	0.52–1.33	0.447
Marital status			
Single	2.82	1.45–5.52	0.002
Married	1.76	0.83–3.75	0.142
Widowed	1.93	0.65–5.74	0.237
Divorced/Separated	Reference	Reference	Reference
Insurance type			
Government	1.55	0.30–7.99	0.598
Private	1.52	0.29–7.82	0.617
Self-pay	1.43	0.23–9.14	0.705
No insurance	Reference	Reference	Reference

OR, odds ratio; CI, confidence interval; OBAT, office-based addiction treatment; SNF, skilled nursing facility; SUD, substance use disorder. Reference group = baseline category used for comparison in the logistic regression model. Missing data for key covariates were rare (<5%) and were handled by complete-case analysis, as sensitivity analyses with simple imputation yielded similar estimates.

Several patient-level characteristics were also associated with an increased risk of readmission. The presence of at least one comorbidity significantly increased the odds of readmission (adjusted OR, 2.41; 95% CI, 1.06–5.45; p = 0.035). Similarly, single marital status was associated with nearly three-fold higher odds of readmission compared with divorced or separated individuals (adjusted OR, 2.82; 95% CI, 1.45–5.52; p = 0.002). Increasing age was modestly but significantly associated with a higher readmission risk (adjusted OR per year, 1.02; 95% CI, 1.00–1.04; p = 0.013). No significant differences were observed according to sex, race, or insurance type.

## Discussion

The findings of this retrospective study demonstrated that hospital-based addiction interventions delivered through the Addiction Consultation Service were associated with significantly lower odds of 30-day readmission among patients with substance use disorders. Patients who received educational counseling or were initiated on pharmacotherapy for opioid or alcohol use disorders had markedly reduced readmission risks compared with those who declined assistance. In contrast, individuals with self-directed discharge experienced a more than threefold higher likelihood, highlighting the critical vulnerability of this subgroup. These results reinforce the importance of structured inpatient addiction services and underscore the need for targeted strategies to prevent premature discharge and strengthen continuity of care following hospitalization. Although the study did not examine patients' engagement in long-term treatment post-discharge, it is reasonable to presume that the observed benefits are partly due to continued treatment adherence.

Our finding that patients with a self-directed discharge had more than threefold higher odds of 30-day readmission aligns with prior evidence, underscoring the elevated risk in this population. Self-directed discharge disrupts the continuity of care, reduces the initiation of indicated treatments, and limits structured linkage to outpatient recovery services, all of which contribute to premature readmission and poorer outcomes. Large cohort studies have consistently demonstrated that self-directed discharge independently predicts increased 30-day readmission and mortality, even after adjustment for comorbidity and illness severity (14). Among individuals with substance use disorders, this effect is amplified, as self-directed discharge often precludes the completion of withdrawal management or initiation of pharmacotherapy for opioid or alcohol use disorders (15). The observed magnitude of risk in our cohort underscores the need for focused in-hospital strategies to *reduce* self-directed discharge, such as early engagement by addiction specialists, rapid initiation of medications for SUD, peer-recovery counseling, and structured “warm handoffs” to outpatient care. By proactively identifying and supporting patients at risk for self-directed discharge, hospital systems may mitigate preventable readmissions and improve the continuity of addiction treatment.

Interventions delivered by the addiction consultation service likely benefit from a structured approach and the expertise of addiction-trained physicians or advanced practice providers who oversee the initiation of medications (16). Although the role of peer recovery specialists was not specifically analyzed, their contributions to education and patient motivation may be important (17). However, the retrospective design of this study limits our ability to capture treatment engagement beyond hospital transitions, such as attendance at outpatient programs, counseling, or participation in support groups. Initiating opioid agonist or alcohol use disorder pharmacotherapy during hospitalization has been shown to enhance stabilization, reduce withdrawal symptoms, and improve patient readiness for continued outpatient engagement (7). These mechanisms likely explain the reduced readmission rates observed in the present study cohort. Prior studies reporting similar benefits underscore the importance of leveraging hospitalization as a ‘reachable moment’ to initiate evidence-based addiction treatment and strengthen the transition of care (18).

Our findings align with those of previous studies demonstrating that hospital-initiated addiction treatment improves outcomes and reduces healthcare utilization (4, 7). These studies support the expansion of addiction medicine consultation services and indicate similar reductions in readmission, overdose risk, and improved treatment linkage. For example, initiating buprenorphine treatment during hospitalization has been associated with higher retention rates in outpatient programs (7).

Canadian data similarly show that the inpatient initiation of opioid agonist therapy is associated with increased outpatient engagement and fewer readmissions (19). Australian studies also support this conclusion; Haber et al. (20) found that integrating inpatient and outpatient addiction services improved adherence and lowered hospital utilization. Together, these international experiences reinforce the generalizability of our findings and highlight the global importance of embedding addiction medicine services into hospital systems to improve patient outcomes.

We did not observe differences in overdose mortality or post-discharge treatment retention; however, these outcomes were not directly measured in our dataset, and the sample size/timeframe may have limited our ability to detect these effects. Although prospective studies are still needed, it is well established that opioid agonist therapy substantially reduces overdose mortality and improves retention (11, 21). Therefore, our findings complement—not contradict—existing evidence by emphasizing inpatient initiation as a critical first step in this continuum. We hypothesized that linkage to comprehensive services at discharge would be associated with fewer adverse outcomes, including recurrence of use, overdose, and hospital readmissions after discharge. While our findings suggest an association with lower odds of readmissions, this study did not examine overdose mortality or treatment retention, which remain important areas for future research.

The significance of this study lies in its potential to guide hospital systems in implementing effective addiction consultation services. By focusing on education and medication initiation, healthcare providers can reduce the burden of re-hospitalization and improve care for individuals with SUD. Future research should aim to quantify the broader societal and economic benefits of these interventions.

### Limitations

This retrospective single-center study has several important limitations, primarily the impact on generalizability and internal validity, as well as limitations on external validity related to patient demographics, local geographic and socioeconomic factors, and institutional practices. Residual confounding from unmeasured variables, such as illness severity, housing instability, or social support, may have influenced the results. Additionally, some heterogeneity likely existed in the content and intensity of the education and referral services. Intervention classification depended on clinician documentation, which may have introduced misclassification bias. We were unable to assess post-discharge medication adherence, outpatient engagement, emergency visits, or overdose events, and our sample size limited the power of the detailed subgroup analyses. Future multicenter studies with longitudinal follow-ups are warranted to address these gaps. Additionally, the timeframe may not fully capture the seasonal or temporal variations in service use. It is also important to note that our data were collected during 2021, a period substantially influenced by the COVID-19 pandemic, which may have affected hospitalization and readmission patterns through disruptions in care delivery, reduced access to outpatient services, and shifts in patient health-seeking behavior. Although this study was conducted during the COVID-19 pandemic, the mechanisms linking inpatient addiction interventions to lower readmission risk, such as improved treatment engagement, stabilization on medications, and enhanced discharge planning, remain applicable to the post-pandemic era. The structural barriers to continuity of care that were evident during COVID (e.g., fragmented transitions, limited outpatient access) continue to exist in many healthcare systems today, suggesting that our findings retain relevance to current practice (22). Next, because of the limited sample size within individual substance categories, we were unable to stratify medication effects by specific pharmacotherapy (e.g., MOUD *vs*. MAUD). Future multicenter analyses with larger cohorts should examine substance-specific outcomes to better delineate these effects on the brain. Finally, readmission data were limited to encounters within the Penn State Health system; thus, patients readmitted elsewhere were not included. This incomplete ascertainment may underestimate the true readmission rates and could introduce bias if out-of-system care differed by intervention exposure.

## Conclusion

Hospital-based addiction interventions, particularly educational counseling and initiation of evidence-based pharmacotherapy, were associated with substantially lower odds of 30-day readmission in patients with substance use disorders. Conversely, patients with self-directed discharge faced a markedly higher risk of early rehospitalization, underscoring the importance of structured engagement and continuity of care during index admission. These findings highlight the critical role of inpatient addiction medicine services in transforming hospitalization into an opportunity for treatment initiation and stabilization. Broader integration of addiction consultation programs that emphasize patient-centered education, timely medication initiation, and coordinated post-discharge linkage has the potential to reduce preventable readmissions and improve long-term outcomes in this high-risk population.

## Data Availability

The datasets presented in this article are not readily available because The dataset contains protected health information derived from electronic medical records and is subject to HIPAA regulations. While de-identified summary data may be shared upon reasonable request, individual-level data cannot be made publicly available due to institutional and legal privacy restrictions. Requests to access the datasets should be directed to pssentongo@pennstatehealth.psu.edu.
